# Biochemical Trade-Offs: Evidence for Ecologically Linked Secondary Metabolism of the Sponge *Oscarella balibaloi*

**DOI:** 10.1371/journal.pone.0028059

**Published:** 2011-11-23

**Authors:** Julijana Ivanisevic, Olivier P. Thomas, Laura Pedel, Nicolas Pénez, Alexander V. Ereskovsky, Gérald Culioli, Thierry Pérez

**Affiliations:** 1 Université de la Méditerranée, Centre d'Océanologie de Marseille, Aix-Marseille Université, CNRS UMR 6540 DIMAR, Station Marine d'Endoume, Marseille, France; 2 Université de Nice-Sophia Antipolis, Laboratoire de Chimie des Molécules Bioactives et des Arômes, CNRS UMR 6001, Institut de Chimie de Nice, Parc Valrose, Nice, France; 3 Université du Sud Toulon-Var, Laboratoire MAPIEM, EA 4323, La Garde, France; J. Craig Venter Institute, United States of America

## Abstract

Secondary metabolite production is assumed to be costly and therefore the resource allocation to their production should be optimized with respect to primary biological functions such as growth or reproduction. Sponges are known to produce a great diversity of secondary metabolites with powerful biological activities that may explain their domination in some hard substrate communities both in terms of diversity and biomass. *Oscarella balibaloi* (Homoscleromorpha) is a recently described, highly dynamic species, which often overgrows other sessile marine invertebrates. Bioactivity measurements (standardized Microtox assay) and metabolic fingerprints were used as indicators of the baseline variations of the *O. balibaloi* secondary metabolism, and related to the sponge reproductive effort over two years. The bioactivity showed a significant seasonal variation with the lowest values at the end of spring and in early summer followed by the highest bioactivity in the late summer and autumn. An effect of the seawater temperature was detected, with a significantly higher bioactivity in warm conditions. There was also a tendency of a higher bioactivity when *O. balibaloi* was found overgrowing other sponge species. Metabolic fingerprints revealed the existence of three principal metabolic phenotypes: phenotype 1 exhibited by a majority of low bioactive, female individuals, whereas phenotypes 2 and 3 correspond to a majority of highly bioactive, non-reproductive individuals. The bioactivity was negatively correlated to the reproductive effort, minimal bioactivities coinciding with the period of embryogenesis and larval development. Our results fit the Optimal Defense Theory with an investment in the reproduction mainly shaping the secondary metabolism variability, and a less pronounced influence of other biotic (species interaction) and abiotic (temperature) factors.

## Introduction

Secondary metabolites have important ecological functions, acting as key mediators in the interaction between organisms and their environment. Their antipredatory and allelopathic roles were exhaustively studied in plant-herbivore and plant-plant interactions [1], whereas their contribution to the functioning of marine biological systems remains poorly explored. However the use of chemical cues is commonplace in marine benthic invertebrates, especially in sessile filter-feeders which are subject to high environmental pressures such as predation, fouling and competition for space and feeding resources [2], [3].

Considering the fact that primary and secondary metabolites have the same chemical precursors (substrates, cofactors), their respective biosynthesis are generally considered to result from a costly trade-off at the biochemical level [4], [5], [6]. Thus, the energy allocated to the secondary metabolism, which mainly dedicated to chemical defenses, is subtracted from the amount of resources available to the remaining physiological functions. Assuming the high cost associated with the production, transport and storage of secondary metabolites, several concepts explaining their evolution and selection have been developed for terrestrial plants and applied to marine organisms. One of the best known models of energy allocation for the production of secondary metabolites is the Optimal Defense Theory (ODT). The ODT asserts that organisms allocate resources to chemical defenses in a way that maximizes fitness [4], [6] and preserves the primary biological functions such as homeostasis maintenance, growth and reproduction. Primary biological functions are mostly determined by seasonal fluctuations of some environmental parameters [7]. Therefore the natural toxicity of an organism and the production of secondary metabolites should vary as a result of a trade-off with primary biological functions, or as a response to biotic and abiotic environmental parameters [8], [9], [10], [11], [12], [13]. Measurements of the bioactivity were often used as indicators in order to determine the baseline variations of the secondary metabolism [13], [14]. Spatial and temporal variations of bioactivity and concentrations of some secondary metabolites were already documented in few sponges, ascidians, and other sessile marine invertebrates [9], [15], [16], [17], [18], [19]. Natural variations were mostly explained by ecological and environmental factors, like biotic interactions [20], [21], [22], [23], habitat type, temperature, depth or salinity [15], [16], [18], [24], [25], [26], whereas the link with the organisms' life cycle (growth, reproduction) or its physiological state was rarely investigated [2], [9], [18], [27], [28]. Regarding secondary metabolites themselves, only few target metabolites were generally studied owing to their valorisation potential, whereas a more holistic view of the metabolome of an organism was not investigated until recently. The tools to carry out such substantial studies have only recently been accessible in a cost effective sense.

Metabolomic approaches can be valuable tools in ecophysiology and ecotoxicology to characterize the physiological responses of an organism at the molecular level, or to learn about the state or condition of the environment (environmental metabolomics and chemical risk assessment) [29], [30]. These approaches are based on a broader metabolome investigation and can provide useful biomarkers of the organisms' health and highlight compounds involved in physiological processes or ecological interactions [31].

Sponges produce a great diversity of secondary metabolites, with original biosynthetic pathways and biological activities [32]. These “chemical weapons” may explain their dominance in some hard substrate communities both in terms of diversity and biomass [33]. This is especially the case in Mediterranean coralligenous formations and semi-dark caves [34], [35], [36] where sponges account for some of the most potent competitors in allelochemical interactions. *Oscarella balibaloi* is a recently described, sciaphilous species which has a very fast colonization trend, and can be often found overgrowing massive sponges, gorgonians and some erected bryozoans [37]. This species is believed to have a great success in some communities of submarine caves because of its highly diverse secondary metabolism compared to other Mediterranean representatives of this sponge group [37], [38].

In this work, we studied the natural variability of the *O. balibaloi* secondary metabolism through the assessment of its bioactivity (or natural toxicity) and its metabolic phenotypes. Our objective was to explain the observed variations by the seasonal fluctuations of the seawater temperature, by the putative influence of different outcompeted species and finally by the sponge reproductive cycle. Thus, our final goal was to test the framework of the ODT by evaluating the potential trade-off between the secondary metabolism (assessed by the bioactivity and metabolic phenotypes) and the primary metabolism (estimated in terms of reproductive effort) with an original biological model.

## Methods

### Model species

**Figure 1:**
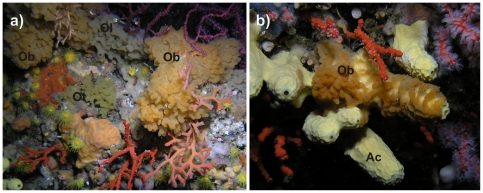
*Oscarella balbaloi in situ.* a) Well developed *Oscarella balibaloi* (Ob) specimen in the vicinity of two common *Oscarella* species: *Oscarella lobularis* (Ol) and *Oscarella tuberculata* (Ot). b) *Oscarella balibaloi* (Ob) growing on demosponge *Aplysina cavernicola* (Ac).

*Oscarella balibaloi* Pérez et al., 2011 is a sciaphilous species dwelling in coralligenous formations or at the entrance of submarine caves generally from 8 to 40 m (**Figure 1**). It is thinly encrusting (from 2 to 5 mm thick), white to orange, very soft and slimy in consistency keeping its turgescency only underwater [37].

### Sampling

The sampling was performed in agreement with French professional diving rules and thanks to collecting permits delivered by the Office of “Préfecture Maritime de Méditerranée”. The monitoring of the reproduction and production of secondary metabolites was performed between November 2007 and November 2009 (two annual cycles) in a population located at 13 m depth in Maire Island, Marseilles (43.2096° N; 5.3353° E). During this period, six different individuals were randomly collected each month along with their substrate. The type of substrate (rock, sponge or cnidarians species) was identified for each individual. A small piece of each individual (about 1 mm^3^) was cut off and fixed for histological study and the assessment of the reproductive effort. The remaining material was carefully separated from the substrate and immediately frozen at -20°C, then freeze-dried and kept at -20°C until the extraction for chromatographic analyses.

### Description of the reproductive cycle

The characterization of the sponge reproductive cycle and the monitoring of the reproductive effort were presented in detail in our publication about the description of *O. balibaloi* [37], new species.

### Secondary metabolites extraction

The freeze-dried samples were ground to obtain a homogenous powder. About 125 mg of this powder was extracted in 5 mL of MeOH/CH_2_Cl_2_ (1∶1, v/v) during 10 min in an ultrasonic bath, this operation being repeated three times. The filtrates of each extraction were combined and evaporated under *vacuum* to dryness. The dry weight of crude extracts did not vary significantly over time. Therefore, the crude extract solution was tested at constant concentration as we assumed that the bioactivity variation was due to the variation in the relative expression level of compounds or their proportion in the overall production.

### Bioactivity assay

The standardized Microtox® bioassay (Microbics, Carlsbad, CA, USA) was used to assess the bioactivity of each *O. balibaloi* individual crude extract (24 months x 6 samples x 3 replicates)**.** The purpose of this test was to give an indication of the sponge bioactivity and assess its intra-specific pattern of variability. We did not intend to give an absolute value of the sponge bioactivity. However, this bioassay correlates well with many other ecotoxicological tests and natural fluctuations of the expression levels of target secondary metabolites (see for instance [13] and [39]). Crude organic extracts were dissolved in artificial seawater and up to 2% of acetone was added to improve the solubilization. Stock solutions were tested at four diluted concentrations. The initial concentration was set at 1 000 µg/mL and a dilution factor of 2 was applied between each following tested concentration. The bioactivity was quantified by measuring the direct effect on the metabolism of the bioluminescent bacterium *Vibrio fischeri* indicated by a decrease in emitted light and expressed as an EC50 value. Thus the lowest EC50 (<350 µg/mL) correspond to the highest crude extract bioactivity, and the highest EC50 (>700 µg/mL) correspond to the lowest bioactivity.

### Metabolic fingerprinting

A selection of 40 sponge extracts, sub-sampled within the periods of the most contrasted mean bioactivities, was analyzed using the metabolic fingerprinting approach developed by Ivanisevic et al. 2011. To cover the period of lowest bioactivity we have chosen all the samples from the period of high reproductive effort: May and June 2008 and 2009 (4 x 6samples + 1 extra sample from June 2009). To cover the period of the highest bioactivity, we have considered 3 samples from each following months: September 2008 and 2009, November 2007, February and December 2008 (5x3samples). However each period considered contained individuals of variable bioactivity, and each sample was analysed in three replicates. Thus, our dataset reflected the intraspecific natural variability with a rather similar number of sponge extracts exhibiting high (EC50<350 µg/mL), low (EC50>700 µg/mL) or moderate (350 µg/mL<EC50<700 µg/mL) bioactivities.

Following the Microtox® bioassay, 5 mL of each aqueous extract were loaded onto conditioned C_18_ SPE (Solid Phase Extraction) cartridge (0.2 g, Phenomenex Strata, Torrance, CA, USA). After desalting with water, organic extracts were eluted with 1 mL of MeOH/CH_2_Cl_2_ (1∶1, v/v). Metabolic profiles were then obtained by on line LC-MS/ELSD (Liquid Chromatography - Mass Spectrometry/Evaporative Light Scattering Detector) analysis using a VWR-Hitachi HPLC system and a Bruker Esquire 3000 Plus mass spectrometer equipped with electrospray ionisation. HPLC separations were achieved on a C_6_-phenyl column (Phenomenex, Gemini, 250 mm × 3.0 mm, 5 µm) using a linear gradient elution of H_2_O/ACN/formic acid from 90∶10∶0.1 (isocratic from 0 to 5 min), to 0∶100∶0.1 (isocratic from 35 to 45 min, flow 0.5 mL/min). The column temperature was maintained at 30°C and the injection volume was set at 20 µL. The mass spectrometry detector (MSD) parameters were set as follows: nebulizer sheath gas, N_2_ (50 psi); dry gas, N_2_ (12 L/min); capillary temperature, 350°C; capillary voltage, 4 000 V in negative ion ESI mode, 4000 V in positive ion ESI mode; lens 1 voltage, −5 V in (+) ESI; lens 2 voltage, −60 V in (+) ESI; isolation width for the MS experiments, *m*/*z* 1.0; collision gas, He; and collision energy, 35%. Data were collected between *m/z* 100 and 800. The ELSD fingerprints were obtained after mobile phase nebulization at 30°C following the evaporation at 50°C. The resulted metabolic fingerprints, ELSD and ESI-MS(+) (Base Peak Chromatogram) raw chromatogram data, were processed by Bruker Daltonics Data Analysis Software version 3.4 (Bremen, Germany). Filtering and baseline corrections for ELSD chromatogram were defined by the following parameters: RT (Retention Time) range 0-50 min, Signal/Noise threshold ≥ 5%, Minimum Relative Area ≥ 1% and Minimum Relative Intensity ≥ 1%. ELSD mode reflects the relative metabolic composition of the sample. The quantification was thus performed on ELSD chromatograms by an integration of the peak area and expressed as a relative peak area (RPA) in % of the whole chromatogram. The compounds identity was assessed by MS (*m/z*) and retention time (RT) data.

### Data Analysis

Non parametric *Kruskal-Wallis test* (KW) was used to analyze the bioactivity variation over time (month), depending on the type of substrate, range in seawater temperature, sponge reproductive stage and sex. Temperature data were kindly provided by the MEDCHANGE project (N. Bensoussan & JC Romano). A mean temperature was calculated for each month that preceded the sampling date by averaging daily temperatures. To distinguish different metabolic fingerprint patterns, we performed a Principal Component Analysis on the data set containing samples of contrasting bioactivity (low *versus* high). Data matrix was composed of detected metabolites as variables and their relative peak area (%) as values.

## Results

### Bioactivity and its temporal variability

**Figure 2:**
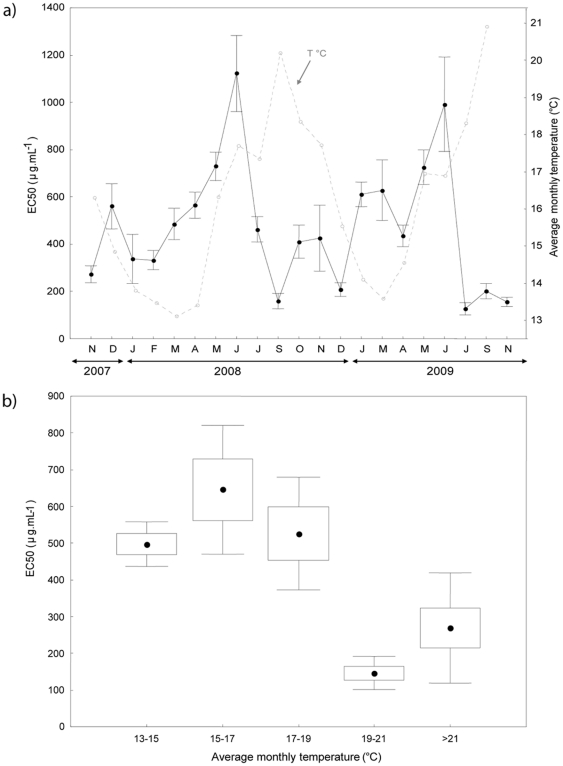
Temporal variation of the bioactivity in relation to the seawater temperature. a) Variation of *Oscarella balibaloi* bioactivity (EC_50_ in µg.mL^−1^) over two years (Kruskal-Wallis test: H KW(20;109)  = 78.86; p<0.0001) in relation to the seasonal fluctuations of the seawater temperature (°C). Vertical bars represent standard errors (Mean ± 0.95 SE). b) Effect of the seawater temperature on the bioactivity of *Oscarella balibaloi*. The bioactivity is indicated in EC50 (µg.mL^−1^). Boxes represent Mean ± 0.95 SE and vertical bars Mean ± 0.95 Confidence Interval. Significant effect indicated by Kruskal-Wallis test: H KW(4;106)  =  27.35, p<0.0001.

The average bioactivity of *Oscarella balibaloi* indicated by the standardized Microtox® assay was 486±32 µg.mL^−1^ (EC_50_ ± SE). It showed a significant seasonal variation following the same trend over the two studied years (2007-2009) (**Figure 2**). The lowest bioactivity was observed in late spring, from May to June with minimal values recorded in June 2008 (EC_50_ = 1123±170 µg.mL^−1^) and June 2009 (EC_50_ = 992±280 µg.mL^−1^). High bioactivities were recorded from mid-summer to late winter (EC_50_ = 383±25 µg.mL^−1^), the maximal values being reached at the end of July and in September 2008 (EC_50_ = 125±26 µg.mL and 159±35 µg.mL^−1^ respectively), then in September and November 2009 (EC_50_ = 201±32 µg.mL^−1^ and 156±35 µg.mL^−1^ respectively).

An effect of the seawater temperature was detected, with a significantly higher bioactivity in warm conditions (**Figure 2**). On average, when the temperature exceeds 19°C, the mean bioactivity is 3–4 times higher than during the coldest periods (<19°C). The lowest mean bioactivity was recorded when the seawater temperature ranged between 15 and 17°C, whereas the highest mean bioactivity was recorded when the temperature ranged between 19 and 21°C (**Figure 2**).

### Metabolic fingerprinting

**Figure 3:**
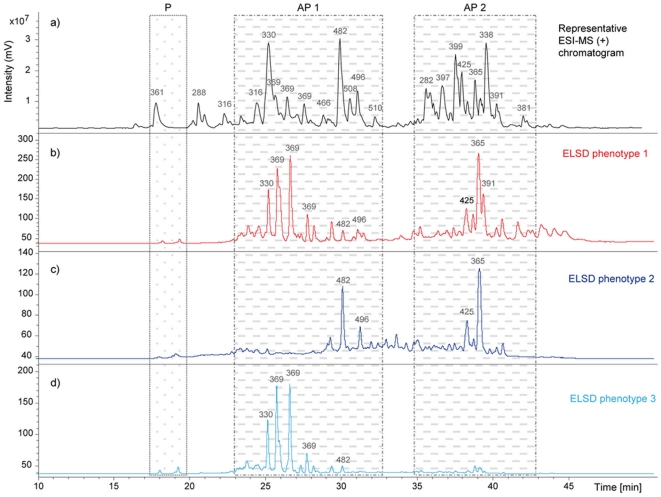
Metabolic fingerprints of *Oscarella balibaloi*. Representative LC-MS chromatogram (a) of *Oscarella balibaloi* and three metabolic phenotypes (**1**, **2** and **3**) indicated by their respective ELSD chromatograms (b, c, d). On the LC-MS and LC-ELSD chromatograms major m/z are indicated above peaks.

**Figure 4:**
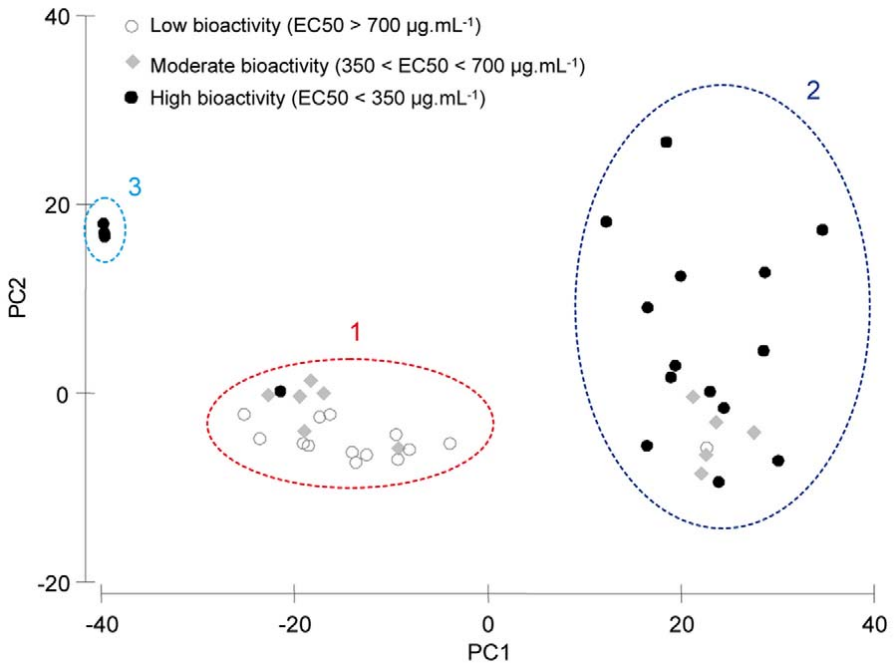
Relationship between the bioactivity and metabolic phenotypes of *Oscarella balibaloi*. The scatterplot of the metabolic fingerprint data set was analyzed by PCA. The PC1 explains 70.2% of variability and PC2 explains 11.3%.

Standardized LC-MS and LC-ELSD fingerprinting enabled the characterization of every analysed sample and gave an overall indication of *O. balibaloi* chemical diversity. Three main groups of compounds were detected (**Figure 3** **,** **Table 1**): the most polar group (P) with RT = 18–20 min, and two rather apolar groups, AP1 with RT = 24–30 min (*m/z* 330, 369, 482 and 496) and AP2 with RT = 37–43 min (*m/z* 425, 365 and 391). The polar metabolites (P) were always less expressed than the apolar groups AP1 and AP2 (**Figure 3** **,** **Table 1**). The highly sensitive LC-MS indicated a high chemical diversity with no qualitative variations of the overall composition in secondary metabolites, whereas HPLC-ELSD profiles were more variable (**Figure 3**). Indeed most of the highly ionisable minor compounds, which were well detected in LC-MS, were in general not detected in LC-ELSD (see for instance compounds *m/z* 288 and 316). Thus the latter method tended to indicate a lower chemical diversity, but also allowed to detect significant quantitative variations of the major secondary metabolites concentrations and to distinguish three main metabolic phenotypes (**Figure 4**). These phenotypes also correspond to distinct bioactivities, the phenotype 1 grouping mainly individuals with low bioactivities, whereas phenotypes 2 and 3 group individuals with high bioactivities (**Figure 4**).

**Table 1:** Secondary metabolite composition of the three distinct metabolic phenotypes of *Oscarella balibaloi*.

	Group of compounds (m/z)								
Metabolic phenotype	Polar (P)		Apolar 1 (AP 1)				Apolar 2 (AP 2)		
	?	?	330	369	482	496	425	365	391
**1**	+	+	+++	+++	++	+	++	+++	++
**2**	+	+	-	-	+++	++	++	+++	-
**3**	+	+	+++	+++	+	-	-	-	-

The expression level of each annotated compound is indicated by the following symbols: “-” in trace, “+” low, “++” medium and “+++” high.

The metabolic phenotype **1** (low bioactivity) is characterized by the detection of all three principal groups of compounds, polar compounds (P) being the less expressed. The metabolic phenotype **2** (mainly high bioactivity) is characterized by the high expression level of two compounds (*m/z* 482 and 496) from the AP1 group that were barely detected in other phenotypes. In this phenotype, other compounds (*m/z* 330 and 369) of the AP1 group were not detected with LC-ELSD, whereas compounds of the apolar group AP2 were well expressed and polar compounds (P) remained barely detected. The metabolic phenotype **3** is represented by only three highly bioactive samples from September 2008. This phenotype is characterized by a low expression level of the P group and a rather high expression level of AP1 (*m/z* 330, 369 and 482), all other compounds not being detected with LC-ELSD (**Figure 3**).

### Bioactivity in relation to biotic interactions

**Figure 5:**
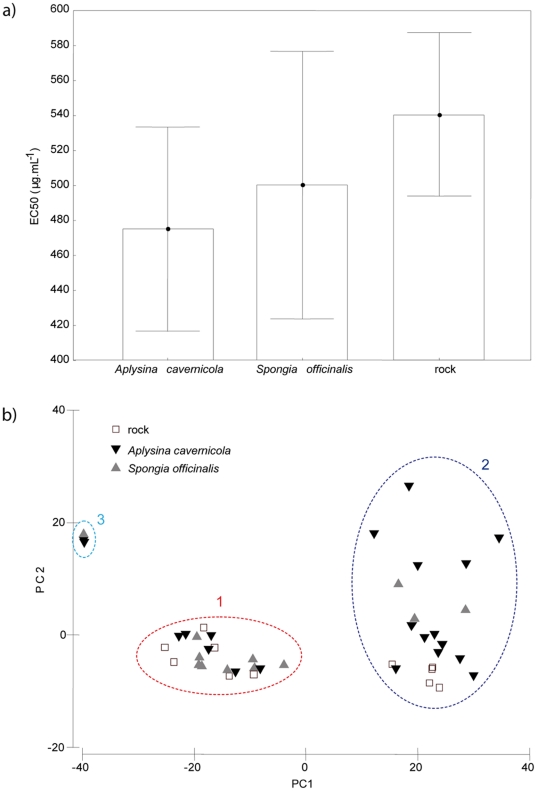
Influence of biotic interactions on *Oscarella balibaloi* bioactivity. a) Interactions with two favorite sponge species, *Spongia officinalis* (n = 32) and *Aplysina cavernicola* (n = 3 4) *versus* rocky substrate (control, n = 26) were tested. Boxes represent Mean ± 0.95 SE and vertical bars Mean ± 0.95 Confidence Interval. Significant effect indicated by Kruskal-Wallis test: H KW(2;90)  =  2.61, p<0.27. b) Relationship between metabolic phenotypes and biotic interactions of *Oscarella balibaloi* represented by the scatterplot of the metabolic fingerprint data set. The PC1 explains 70.2% of variation and PC2 the additional 11.3%.

*Oscarella balibaloi* was often sampled overgrowing two demosponge species, *Spongia officinalis* and *Aplysina cavernicola*. Although the substrate effect on the *O. balibaloi* bioactivity was not significant (H, KW (2; 90)  =  2.61, p = 0.27) (**Figure 5**), the highest bioactivities were recorded when the sponge was overgrowing *A. cavernicola*. Furthermore, the metabolic phenotype **1** corresponds to individuals collected on every type of substrate (*A. cavernicola*, *S. officinalis* and rock) whereas the metabolic phenotype **2** is mainly represented by individuals overgrowing *A. cavernicola* (**Figure 5**). Among the three samples corresponding to the phenotype **3**, two were collected on *A. cavernicola* and one on *S. officinalis.*

### Bioactivity variation in relation to the sponge reproductive cycle

**Figure 6:**
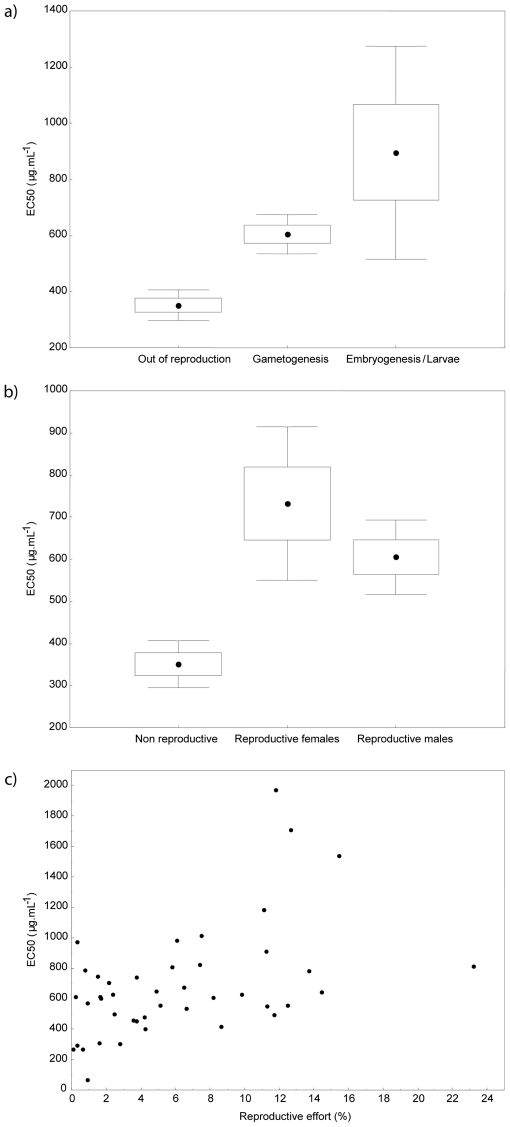
Influence of sponge reproductive status on the bioactivity. a) Variation of *Oscarella balibaloi* bioactivity (EC50 in µg.mL^−1^) according to reproductive stage. The effect of significance is given by Kruskal-Wallis test: H KW(2;109)  =  31.48, p<0.001. b) Variation of *Oscarella balibaloi* bioactivity (EC50 in µg.mL^−1^) according to sponge sexe. The effect of significance is given by Kruskal-Wallis test: H KW(2;109)  =  31.44, p<0.0001. c) Correlation between *Oscarella balibaloi* bioactivity (EC50 in µg.mL^−1^) and its reproductive effort (%): R = 0.49, p = 0.0008. Boxes represent Mean ± 0.95 SE and vertical bars Mean ± 0.95 Confidence Interval.

**Figure 7:**
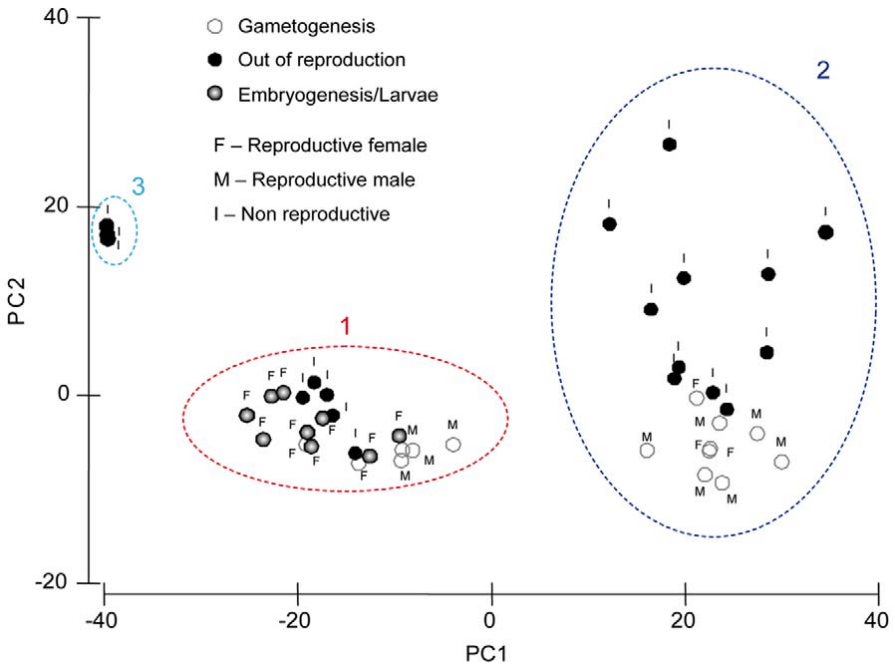
Relation between metabolic phenotypes and sponge reproductive status. The scatterplot of the metabolic fingerprint data set was analyzed by PCA. The representation factor is the reproductive stage with the indication of the sponge sexe. The PC1 explains 70.2% of variation and PC2 the additional 11.3%.

*Oscarella balibaloi* is a gonochoric and ovoviviparous species. Oocytes were observed almost all year long whereas spermatocytes only occurred between March and early July. The embryonic development was observed from April to mid-August. Over the studied period (2007-2009) the female reproductive effort reached its maximal values in June (23.4% in 2008 and 15.3% in 2009) during the embryogenesis and larval development. Overall, the mean annual female reproductive effort (6.32±1.5%) was two times higher than the male reproductive effort (3.58±0.9%) which also reached its maximal values in June. The bioactivity of *O. balibaloi* varied significantly in relation to the sponge life cycle and different reproductive stages (gametogenesis *versus* embryonic and larval development) (**Figure 6**). It slightly decreased from April to June, the minimal bioactivity coinciding with the period of embryogenesis and larval development (**Figure 2** **and** **6**). In general the bioactivity is negatively correlated to the reproductive effort (**Figure 6**). Coherently, we observed a significant lower bioactivity in reproductive sponges, and in females, which exhibited in overall the highest reproductive effort (**Figure 6**). Thus all females exhibited the metabolic phenotype **1**, whereas the most bioactive metabolic phenotypes **2** and **3** were mainly represented by non-reproductive sponges (**Figure 7**). Finally, depending on their reproductive effort, male sponges were distributed among the chemical phenotypes **1** and **2**.

## Discussion

*Oscarella balibaloi* has a bioactivity which is in average 2 times lower than *O. tuberculata,* the most bioactive Homoscleromorpha sponge studied so far using the same standardized method [38]. These two *Oscarella* species harbor a significantly divergent diversity of secondary metabolites, as indicated by their metabolic fingerprints, and thus belong to two well separated clades of Homoscleromorpha without skeleton [38]. The divergence in metabolic diversity could be associated with different symbiotic microorganism assemblages harbored by these two species. The symbiotic bacteria seem to be species specific [40], [41], [42], [43], [44] and their morphotypes are also used as cytological markers for species determination in the absence of other morphological characters [45]. As the symbiotic community is species-specific and rather stable in time and space, the compounds that might be produced by these components of the sponge holobiont are also supposed to be species specific. Considering our extractive and analytical procedures, *O. balibaloi* harbors higher metabolite diversity than *O. tuberculata* [38], but it does not produce three of its major compounds, the lysophosphatidylethanolamine C20:2 and the two 5-alkylpyrrole carboxaldehydes [46]. A lysophospholipid lyso-PAF, recently identified in *O. tuberculata* (C16, *m/z* = 482, RT = 30 min), is probably also present in *O. balibaloi* together with assumed close derivatives with additional methylene units (*m/z* 496, 510) (**Figure 6**).

*Oscarella balibaloi* bioactivity showed a significant annual periodicity with minimal values in late spring, from May to June, and maximal values throughout late summer, autumn and winter periods. Seasonal variations of bioactivity and secondary metabolites production seem to be commonplace in benthic marine invertebrates in the Mediterranean Sea [2], [9], [13], [18], [19], [26], [47]. A seasonality, with higher bioactivities in late summer and autumn was observed for *Crambe crambe* and *O. tuberculata* among other sponge species from Mediterranean benthic communities [19], [26]. On the other hand, two other common Mediterranean species, *Agelas oroides* and *Petrosia ficiformis*, displayed also a higher bioactivity in winter whereas in summer the bioactivity was lower or absent [18]. It was often hypothesized, but never proved, that variations of sponge chemical defenses can be driven by internal physiological factors, thus resulting from trade-offs with primary biological functions such as growth and/or reproduction [2], [9], [10], [18], [19], [27], [48], [49]. These “internal” factors may act together with other biotic factors, such as competition for space [20], [21], [22], [23], and a number of abiotic factors (i.e. seawater temperature and salinity, light intensity and/or nutrient bioavailability) [15], [16], [48]. 

In our two-year study, we detected a significantly higher bioactivity during the warmest periods, and more generally when sponges were subjected to seawater temperature exceeding 19°C. Before the warmest period of the year, a progressive decrease in bioactivity was observed. This could indicate that temperature has an indirect effect on the secondary metabolism and mainly triggers other processes, like the sponge reproduction [19]. In early spring, *O. balibaloi* undergoes gametogenesis and the occurrence of these first reproductive events may explain the decrease in bioactivity observed while the seawater temperature is rising.

*Oscarella balibaloi* has a seasonal reproductive cycle with a highest reproductive effort from May to the end of June, coinciding with the seawater temperature rising in spring. The period of high reproductive investment (up to 23% of female tissue) corresponds to stages of embryogenesis and larval development. Seasonal reproduction patterns are common in temperate habitats [10], [50], [51] and could be related primarily to metabolic constraints of the thermal regime and nutrient bioavailability [7], [27]. Moreover a seasonal bioactivity pattern, with the lowest bioactivities during the sponge reproduction, suggests a trade-off between the resources dedicated to the reproductive processes and the production of secondary metabolites. Indeed the reproduction and the production of secondary metabolites compete for the same limited resources, and thus an increase of investment in reproduction is likely at the expense of the production of secondary metabolites. As the release of larvae in the late summer, when the temperature reaches its maximum, comes also with the end of the energy investment in reproduction, a switch towards a greater production of secondary metabolites is then observed. A major influence of the reproductive cycle could explain why the bioactivity reached its maximum at this period of the year and then remained high throughout the autumn and winter in cold conditions.

Clear evidences of trade-off between the reproduction and the production of secondary metabolites in marine invertebrates are scarce and our findings confirm the first hypothesis by Lopez-Legentil et al. [2]. In a temporal study of the production of the alkaloid ascididemin, the major antipredatory molecule of the ascidian *Cystodytes dellechiajei*, these authors recorded the lowest expression levels during the oocytes maturation and the highest after the larvae release. Besides this work, the only indications of trade-off originate from studies of temporal variability patterns of other biological functions. For instance, the resource allocation concept between the growth and production of chemical defenses was described in the well studied Mediterranean sponge species *C. crambe* [10], [21] and for a group of common sponges of Caribbean coral reefs [27]. Although a significant negative relationship between the growth rate and the bioactivity was reported for *C. crambe* [10], only a low level of correlation was found for Caribbean sponges. In the latter case, the authors stated that other resource trade-offs could obscure the existing relationship, one hypothesis being a putative influence of the reproduction processes [27].

Encrusting sponges such as *O. balibaloi* are often considered as superior competitors [52], the overgrowth being a widespread mechanism employed to compete with neighbors for space [53]. We observed a weak increase in the bioactivity of *O. balibaloi* when growing on other sponges. *Oscarella balibaloi* habit is even more curious considering the “chemical weapons” of the outcompeted species. For instance, *A. cavernicola* contains high concentrations of bromotyrosine derivatives which are well known for their antimicrobial and cytotoxic activities and for their repellent properties against predators and epizoans [54], [55], [56], [57]. Even so, some necroses of *A. cavernicola* tissues are often observed when *O. balibaloi* is overgrowing its surface (personal observation). However, such necroses were rarely observed among other out-competed species which suggest that *O. balibaloi* can co-exist as an epizoic species on living substrates without production of antagonistic allelochemicals. This could explain the lack of statistical support for the trend we observed and confirm several previous studies which demonstrated that the bioactivity was not affected by the presence of competitors [22], [23], [58]. It seems that competition for space does not significantly influence the bioactivity and that other factors have a prevailing influence on the high inter-individual variability often observed.

As indicated by our rapid assessment method, *Oscarella balibaloi* harbours a high diversity of secondary metabolites differing clearly from the metabolic fingerprints of other *Oscarella* species [37], [38]. Although non exhaustive, our extractive and chromatographic procedures gave an overview of the general metabolic composition of an individual. The strength of this approach, combining two types of detection (LC-MS and LC-ELSD), lies in its usefulness to assess and compare inter- and intra-specific variability of the metabolic composition of marine organisms. After our application of LC-MS fingerprints to the Homoscleromopha chemical taxonomy [38], we show that LC-ELSD can be a good tool to study the natural variability of the most expressed part of the *O. balibaloi* metabolome. More generally, this method can link secondary metabolite changes to both biological causes and physiological consequences [29], [59]. In our case it allowed to discriminate several metabolic phenotypes coherently assigned to different categories of individuals. The less bioactive phenotype **1** is mainly represented by reproductive females harboring embryos or larvae, whereas the most bioactive phenotypes **2** and **3** are represented by non-reproductive individuals or males with low reproductive efforts.

When *O. balibaloi* was experiencing embryogenesis, a bioactivity decline and a high expression of several compounds from the AP1 group (*m/z* = 330 and 369) were recorded. The high expression of these compounds is associated to females with high reproductive effort, thus suggesting a putative role in the sponge embryogenesis. However their presence in three non-reproductive and highly bioactive sponges of the metabolic phenotype **3** is intriguing. As often suggested for secondary metabolites, these compounds might also have an additional defensive role and thus multiple functions [3]. Furthermore, the lyso-PAF already identified in *O. tuberculata* seems to be highly expressed in individuals of the chemical phenotype 2 (**Figure 6** **).** As this molecule was already reported for its antifouling properties [60], [61], we can hypothesize that it significantly contributes to the detected bioactivity in *O. balibaloi* as well.

Above all the other studied factors, the reproductive output influences the bioactivity and the production of secondary metabolites. We observed an allocation pattern between the reproductive effort and secondary metabolism that can be related to the ODT. The ODT hypothesizes that organisms allocate defenses in a way that maximizes fitness, and mainly focuses on the putative influence of predation on the production of secondary metabolites. Taking into account that defenses are costly when enemies are absent, their production is generally adjusted in relation to the predation pressure. For instance, seaweeds can allocate more resources to chemical defenses when the herbivores pressure is high [62]. It is also well known that herbivores act as a driving force in the evolution of the secondary metabolism [1]. In sciaphilous communities, the predation pressure is lower [63] and a lot of sciaphilous sponges do not have predators. The competition for space in these communities is rather high and could be a major driving force in the evolution of secondary metabolite diversity. However, a number of papers demonstrated that the presence of competitors does not significantly influence the production level of secondary metabolites [22], [23], [58]. Thus assessing the putative influence of external biotic factors on the production of chemical defenses would require dissecting the combined influence of a high diversity of competitive interactions that occur in those communities. Our study demonstrated that internal factors, such as the investment in the reproduction, can have a prevailing effect on the secondary metabolism by triggering the resource allocation. These results fit the Optimal Defense Theory, but the combined influence of numerous internal and external biotic and abiotic factors on the secondary metabolism make difficult the assessment of its energetic cost. In this study we did not explore the possible influence that a varying symbiont load could have on the secondary metabolite production. However, the abundance and diversity of symbiotic microorganisms that have co-evolved and live in close association with sponge seem to be species specific [43] as mentioned earlier. Numerous studies have shown that the structure of the symbiont community is largely independent of the surrounding seawater [42], [64], [65], [66]. Moreover, some investigated, intraspecific, temporal and spatial variations of the bacterial association appeared rather low [40], [67]. Therefore, we believe that symbiotic bacteria cannot contribute significantly to the variability of sponge secondary metabolism. The next step of our on-going research will be the structural elucidation of the characteristic compounds of each metabolic phenotypes and a deeper investigation of their origin (host organism and/or symbionts), their biological and ecological function.
